# Supplementary material for: Mutations in PCYT2 disrupt etherlipid biosynthesis and cause a complex hereditary spastic paraplegia
Source: Brain. 2019 Oct 22;142(11):3382–97. doi: 10.1093/brain/awz291 (PMC6821184; doi:10.1093/brain/awz291)
Supplement: awz291_Supplementary_Data [file awz291_supplementary_data.zip › awz291-Suppl_data/Supplementary_Data4.zip!/Supplementary information 4 - plasma lipidomics.zip!/Plasma lipidomics/Control vs Patient1/Statistical plots and data/PA species/Random forest.pdf]

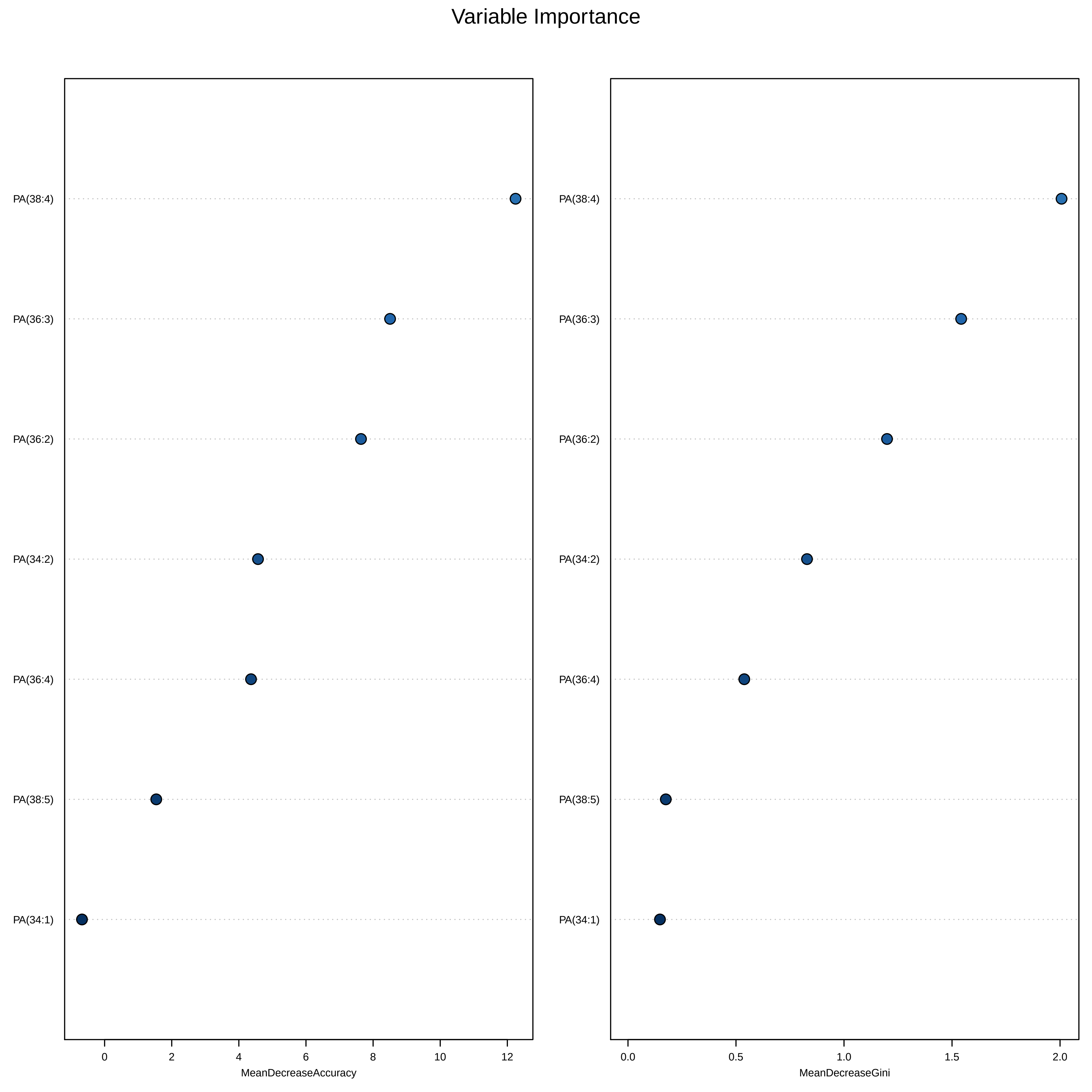

Variable Importance
PA(38:4)
PA(36:3)
PA(36:2)
PA(34:2)
PA(36:4)
PA(38:5)
PA(34:1)
0
2
4
6
8
10
12
MeanDecreaseAccuracy
0.0
0.5
1.0
1.5
2.0
MeanDecreaseGini
